# Supplementary material for: Efficient Production of Self-Assembled Bioconjugate Nanovaccines against Klebsiella pneumoniae O2 Serotype in Engineered Escherichia coli
Source: Nanomaterials (Basel). 2024 Apr 21;14(8):728. doi: 10.3390/nano14080728 (PMC11054253; doi:10.3390/nano14080728)
Supplement: Supplementary file 1 [file nanomaterials-14-00728-s001.zip › nanomaterials-2925231-supplementary.pdf]

Supplementary Table S1 Primers used in this study.

Primer	Sequence
G33A-F	CACCGAGAGCCTGGCAGCCAAGCGCGAGAT
G33A-R	GCTGCCAGGCTCTCGGTGTAGCTAAAAATT
G33D-F	AGCTACACCGAGAGCCTGGCAGACAAGCGCGAGATGGCCAT
G33D-R	ATGGCCATCTCGCGCTTGTCTGCCAGGCTCTCGGTGTAGCT
G33E-F	GCTACACCGAGAGCCTGGCAGAGAAGCGCGAGATGGCCAT
G33E-R	ATGGCCATCTCGCGCTTCTCTGCCAGGCTCTCGGTGTAGC
G33F-F	ACCGAGAGCCTGGCATTCAAGCGCGAGATGGCC
G33F-R	GGCCATCTCGCGCTTGAATGCCAGGCTCTCGGT
G33H-F	AGCTACACCGAGAGCCTGGCACACAAGCGCGAGATGGCCA
G33H-R	TGGCCATCTCGCGCTTGTGTGCCAGGCTCTCGGTGTAGCT
G33I-F	ACACCGAGAGCCTGGCAATCAAGCGCGAGATGGCCATCATC
G33I-R	GATGATGGCCATCTCGCGCTTGATTGCCAGGCTCTCGGTGT
G33K-F	GCTACACCGAGAGCCTGGCAAAGAAGCGCGAGATGGCCAT
G33K-R	ATGGCCATCTCGCGCTTCTTTGCCAGGCTCTCGGTGTAGC
G33L-F	ACACCGAGAGCCTGGCACTCAAGCGCGAGATGGCCATCAT
G33L-R	ATGATGGCCATCTCGCGCTTGAGTGCCAGGCTCTCGGTGT
G33M-F	GCTACACCGAGAGCCTGGCAATGAAGCGCGAGATGGCCAT
G33M-R	ATGGCCATCTCGCGCTTCATTGCCAGGCTCTCGGTGTAGC
G33N-F	GCTACACCGAGAGCCTGGCAAACAAGCGCGAGATGGCCAT
G33N-R	ATGGCCATCTCGCGCTTGTTTGCCAGGCTCTCGGTGTAGC
G33P-F	GCTACACCGAGAGCCTGGCACCAAAGCGCGAGATGGCCAT
G33P-R	ATGGCCATCTCGCGCTTTGGTGCCAGGCTCTCGGTGTAGC
G33Q-F	CTACACCGAGAGCCTGGCACAAAAGCGCGAGATGGCCATC
G33Q-R	GATGGCCATCTCGCGCTTTTGTGCCAGGCTCTCGGTGTAG
G33R-F	TACACCGAGAGCCTGGCACGCAAGCGCGAG
G33R-R	GTGCCAGGCTCTCGGTGTAGCTAAAAATTT
G33S-F	GCTACACCGAGAGCCTGGCATCAAAGCGCGAGATGGCCAT

G33S-R	ATGGCCATCTCGCGCTTTGATGCCAGGCTCTCGGTGTAGC
G33T-F	CTACACCGAGAGCCTGGCAACCAAGCGCGAGATGGCCATC
G33T-R	GATGGCCATCTCGCGCTTGGTTGCCAGGCTCTCGGTGTAG
G33V-F	ACACCGAGAGCCTGGCAGTCAAGCGCGAGA
G33V-R	ACTGCCAGGCTCTCGGTGTAGCTAAAAATT
G33W-F	GCTACACCGAGAGCCTGGCATGGAAGCGCGAGATGGCCAT
G33W-R	ATGGCCATCTCGCGCTTCCATGCCAGGCTCTCGGTGTAGC
G33Y-F	GCTACACCGAGAGCCTGGCATACAAGCGCGAGATGGCCAT
G33Y-R	ATGGCCATCTCGCGCTTGTATGCCAGGCTCTCGGTGTAGC
